# Retrospective Comparative Analysis of Clinical and Functional Outcome After Arthroscopic Bankart Repair using All-Suture Anchor and Metal Anchor

**DOI:** 10.5704/MOJ.2403.002

**Published:** 2024-03

**Authors:** V Jain, H Gupta, N Mehta, D Joshi, Ajay, H Kataria

**Affiliations:** Sports Injury Centre, Vardhman Mahavir Medical College and Safdarjung Hospital, Delhi, India

**Keywords:** bankart lesion, shoulder dislocation, suture anchor

## Abstract

**Introduction:**

Both knotted all suture anchors and metal anchors are used for arthroscopic Bankart repair. We retrospectively evaluated and compared clinical and functional outcomes after arthroscopic Bankart repair using the knotted all-suture anchors and knotted metal anchors.

**Materials and methods:**

In a retrospective cohort analysis, patients who underwent arthroscopic Bankart repair without any concomitant additional lesion repair using either all-suture anchors or metal anchors, between January 2015 and May 2018 were identified. Their pre- and post-operative functional and clinical outcomes were compared using Rowe and WOSI scores. The recurrence rate in the two groups was also compared.

**Results:**

A total of 41 patients in all suture anchors group and 47 in the metal anchors group were identified as per inclusion and exclusion criteria. The demographic profile of both groups was comparable. There was no significant difference in clinical and functional outcome between the two suture anchor groups as per Rowe (pre-operative 40.13+6.51 vs 38.09+6.24 and post-operative 2 years 93.28+7.09 vs 92.55+9.2) and WOSI (pre-operative 943.05+216.64 vs 977.55+165.46 and post-operative 2 years 278.21+227.56 vs 270.94+186.25) scores. There was a significant improvement in both the groups between preoperative and post-operative ROWE and WOSI scores at 6 months and 2 years follow-up as compared to pre-operative scores (p<0.001). Re-dislocation rates were also comparable (4.8% vs 6.3%).

**Conclusion:**

All-suture anchors showed comparable clinical and functional results as the metal anchors for arthroscopic Bankart repair at two-year follow-up.

## Introduction

Arthroscopic Bankart repair has become the standard of care for cases of shoulder instability. The evolution of surgical techniques, implants and instrumentations have contributed to the success of arthroscopic management of such cases^1-3^. Metallic suture anchors were first used in the early 1990s^4^. Over a period of time, different anchors like bioabsorbable, PEEK, biocomposite etc were introduced, the most recent being all-suture anchors. All types of material used for these anchors have reported different complications, necessitating constant evolution of material5.

All-suture anchors have the benefit of less bone removal and occupy less volume, this facilitates the insertion of more anchors, resulting in more robust labral repair and easier revision in case of failure^5-9^. They do not interfere with MR imaging, have uniform implant construct and do not cause problems seen in biocompatible anchors due to differential absorption of their constituents^6,8^. Biomechanically they have been found to be equivalent to biocomposite and bioabsorbable suture anchors^6,9^. There are a few studies on the clinical outcome of all-suture anchors but there is a paucity of clinical studies on the comparison of the all-suture anchor with other suture anchors^10^.

Metal suture anchors were the earliest to be introduced and therefore have been the longest in use. They were associated with complications like migration, cartilage erosion, difficulty in revision and MR imaging^5,11-14^. Still, they are one of the commonest and most economical suture anchors in use^15^. Different comparative studies have reported better or comparable clinical functional outcomes of the knotted metal anchors in comparison with biocompatible anchors^14,16-19^.

The purpose of this study was to retrospectively evaluate and compare clinical and functional outcomes after arthroscopic Bankart repair with knotted all-suture anchors and knotted metal anchors in cases of recurrent dislocation shoulder. The outcome measures were Rowe score, Western Ontario Shoulder Instability Index (WOSI) and re-dislocation rate. We hypothesised that the outcomes after arthroscopic Bankart repair would be comparable between the knotted all-suture anchors and knotted metallic suture anchors.

## Materials and Methods

This was a retrospective cohort study on patients who underwent arthroscopic Bankart repair using either knotted all-suture anchors or metal suture anchors from January 2015 to May 2018 in this centre. Permission was obtained from Institutional Ethics Committee (IEC).

The inclusion criterion was isolated Bankart lesion diagnosed on arthroscopic examination which was repaired using either all-suture anchors or metal anchors and minimum post-operative follow-up of two years. Revision procedures, Hill-Sachs lesion requiring remplissage, significant glenoid bone loss (greater than 20%), multidirectional instability, any other concomitant injury requiring repairs such as posterior labral tear, superior labral anterior to posterior (SLAP) tear or rotator cuff tear, use of two or more different types of anchors and any patient with missing data were excluded.

Although the study design was retrospective, the patients were identified from a prospectively collected database containing diagnosis and procedural information from patient admission, discharge, operation theatre and follow-up clinic records. The evaluation was done using pre and postoperative Rowe score, Western Ontario Shoulder Instability Index (WOSI), change in WOSI score (pre-operative score subtracted from post-operative score) and re-dislocation rate.

Change in WOSI score was calculated for each patient and evaluated whether it was more or less than the Minimal clinically important difference (MCID), which was taken to be 220^20^. Information was entered on case to case basis by two authors. For patients who were not able to come for at least a single follow-up after the completion of two years post-operative period, they were contacted on phone and were asked to return Rowe and WOSI questionnaires via the digital medium. Data and statistical analysis were done by two authors, different from the one who entered the data initially, to minimise bias. They were blinded to the group of patients.

Pre-operative workup included clinical examination, MRI of the concerned shoulder, pre-anaesthetic work-up and shoulder function assessment using Rowe score and WOSI score. Patients who underwent arthroscopic Bankart repair using all-suture anchors were put in group A while those in whom metal anchors were used were put in group B.

**Fig 1:**
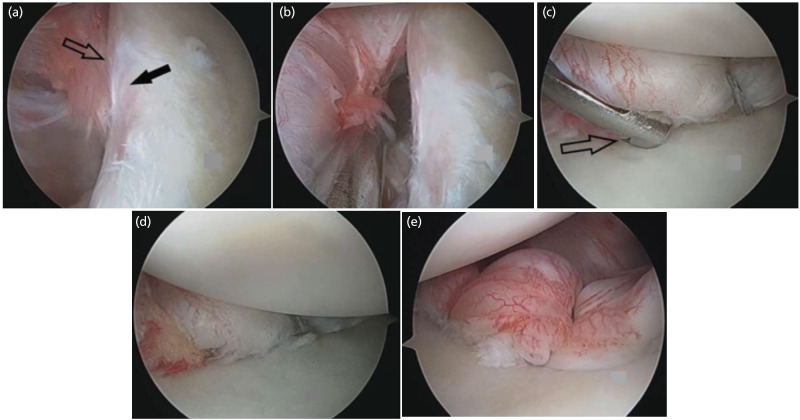
(a) Bankart lesion. Solid arrow marks anterior glenoid margin and open arrow marks rolled up inferiorly displaced labral tissue. (b) Labral release from anterior inferior glenoid. (c) Second anchor placement with specific drill sleeve (open arrow) for all suture anchor. (d) Final repair using all suture anchor. (e) Another case of all suture anchor, final repair.

All the cases were done by four different surgeons trained in shoulder arthroscopy at a single centre with a minimum experience of five years in shoulder arthroscopy. Patients were put under general anaesthesia in the lateral decubitus position. A standard posterior arthroscopic viewing portal was first made and diagnostic arthroscopy of the shoulder joint was performed. Evaluation of glenoid and humeral articular surfaces; rotator interval; anterior, superior, and posterior labrum; superior, middle, and inferior glenohumeral ligaments; biceps and subscapularis tendons; and the axillary pouch was done. Anterior inferior and anterior superior portals were created and a shoulder cannula was inserted in each portal. Measurements of the anteroposterior glenoid and Hill Sach’s lesion were done with a graduated probe to assess the need for remplissage. The anterior labral tear was probed and labral tissue adequately mobilised from the scapular neck from the anterior and anteroinferior margin, till the labrum started floating up at the level of glenoid and underlying subscapularis muscle was exposed with a tissue liberator (Fig. 1a, b) The anterior and anteroinferior scapular neck and glenoid margin were roughened with a rasp.

In the case of all-suture anchors, a 25° curved drill guide was used for inferior most anchor placement and a 12° curved drill guide was used for subsequent anchor placement (Fig. 1c). For, superior anchor placement, a straight drill sleeve was used. A flexible self-centring 1.4mm drill which drills to 20mm depth was used for creating a bone socket for anchor placement. A 1.4mm all-suture anchor with 1 strand # 2 polyethylene fibre [ICONIX, Stryker Corporation, Colorado, USA] was used for labral repair. After insertion in the bony socket, the suture was pulled back and toggled until it was deployed completely.

In the case of metal anchors, a straight fish mouth drill guide was used for drilling a pilot hole of 11.7mm. A 2.8mm titanium metal anchor [FASTak II, Arthrex, Naples, USA] was deployed in the pilot hole and the anchor sleeve was removed.

A 25° curved tissue penetrator and suture shuttle device was used to pass anchor suture threads through the labral ligamentous complex and the same was tensioned and secured with sliding locking knots, shifting the whole labral ligamentous complex superiorly and laterally. In all cases at least three suture anchors were inserted for labral repair (Fig. 1d, e). Insertion of any additional anchor was at the surgeon’s discretion according to intra-operative assessment.

Post-operatively, all patients were placed in an arm sling for six weeks. They were started on pendulum exercises, and elbow, wrist, and hand movements from the first postoperative day. Suture removal was done at two weeks. Patients visited the outpatient physiotherapy department at two weeks for reassurance and reassessment and every two to three weeks thereafter. Shoulder flexion and abduction were allowed till 300 and incrementally increased by 300 every 2 weeks. The sling was removed at six weeks. External rotation beyond neutral and strengthening was started after 8-10 weeks post-operatively. It was usually aimed to achieve a full range of motion at around 12-14 weeks post-operatively, but aggressive external rotation stretching was avoided. Patients were advised to avoid contact sports participation till six months post-operatively. Patients had post-operative clinical follow-ups at six weeks, three months, six months, one year and at two years by four primary surgeons. Patients were assessed for range of motion and any episode of instability or dislocation. Shoulder function assessment was done at six months and two years post-operatively with Rowe score and WOSI.

The mean and standard deviation (SD) for the quantitative functional scores were calculated for each group. Improvements in Rowe and WOSI scores at six months and two years post-operatively were compared to the preoperative scores using paired t-test in each of the two groups separately. The average MCID and percentage of patients having a change in score less than MCID was determined for both groups. All the quantitative values were in mean + SD and range mentioned. Confidence interval (CI) was determined for pre and post-operative functional scores and change in score for WOSI. Independent two sample t-test or Mann-Whitney test was used for comparing the difference in clinical outcome scores and MCID between the two groups. Re-dislocation rate between the groups was compared by Fischer exact test. P<0.001 was considered statistically significant.

## Results

A total of 161 patients were found in the database who had undergone arthroscopic Bankart repair in the study period. There were 57 and 63 patients in whom all-suture anchors and metal anchors were used, respectively. After considering the exclusion criterion there were 41 patients in group A and 47 patients in group B (in group A 16 patients and in group B 10 patients were excluded on account of concomitant SLAP, remplissage and posterior labral repair. Further, in group B, data was incomplete in three patients and two different types of anchors were used in three patients).

The demographic details of the two groups and the mean and SD of pre-operative functional scores are given in Table I. These baseline characteristics were not significantly different between the two groups. There was no missing data from the patients included in the study.

**Table I:** Baseline characteristics of patients.

	Group A (n=41)	Group B (n=47)	p-value
Age at surgery (years) (mean+SD) (Range)	26.43+8.24 (18-52)	25.85+7.06 (17-51)	0.72
Sex (Male: Female)	37:4	43:4	1
Affected side (Right: Left)	28:13	30:17	0.65
Involved Hand (Dominant: Non-dominant)	29:12	32:15	0.78
The average number of dislocations pre-operatively (Range)	4.51 (2-8)	4.35 (2-8)	0.66
Average time since 1st dislocation to surgery (months)	26.29 (6-60)	28.08 (4-72)	0.61
Competitive Sportsperson (number of cases)	15	17	0.96
Average follow-up (months) (mean+SD) (Range)	35.12+7.99 (24-50)	57.15+8.90 (33-64)	<0.001
Number of re-dislocation	2 (4.8%)	3 (6.3%)	1

SD= Standard deviation

**Fig 2:**
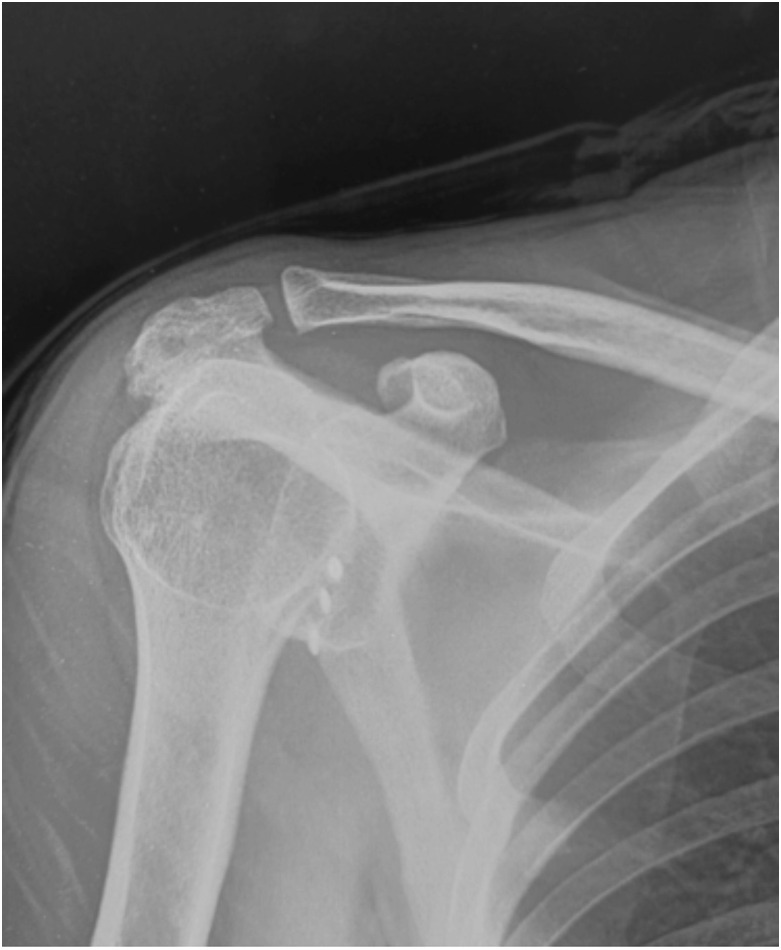
Radiograph image of Bankart repair using metal anchor.

There was a significant improvement in post-operative ROWE and WOSI scores at six months and two years follow-up as compared to pre-operative scores in both group A and group B (p<0.001 each). There was no significant difference in post-operative ROWE and WOSI scores at six months and at the last follow-up as well as change in WOSI (pre-operative and two-year follow-up) between the two groups (Table II). In all, 123 all-suture anchors were used in group A and 140 in group B. Radiograph finding at two-year follow-up were available in 30 patients in the all-suture anchors group and 32 patients in the metal anchors group (Fig. 2). None of the radiographs in the groups revealed any significant findings.

**Table II:** Clinical and functional outcomes.

	Group A	Group B	Comparison of Group A vs B	
			p-value	95% CI for difference in scores
ROWE Score, mean+SD (Range):				
(1) Pre-op	40.12+6.17 (25-45)	38.09+5.66 (25-45)	0.1	-0.43 to 4.58
(2) 6 months post-op	83.46+9.67 (70-95)	85.21+10.53 (55-95)	0.4	-2.55 to 6.05
(3) 2-year post-op	90.85+12.59 (40-100)	92.55+9.2 (40-100)	0.6	-2.93 to 6.33
WOSI, mean+SD (Range):				
(1) Pre-op	945.97+213.95 (616-1446)	977.55+165.46 (632-1319)	0.4	-48.95 to 112.10
(2) 6 months post-op	445.97+152.25 (159-791)	475.83+144.82 (229-933)	0.3	-33.14 to 92.86
(3) 2-year post-op	303.60+248.14 (54-1321)	284.21+197.87 (49-874)	0.9	-75.20 to 113.98
Change in WOSI score at 2 years compared to pre-op score, mean+SD (Range)	642.37+237.65 (124-1238)	678.90+200.40 (91-982)	0.4	-56.30 to 129.35
Percentage of patients with WOSI change less than MCID	7.31%	4.25%	1.0	

Abbreviations - Pre-op: pre-operative, post-op: post-operative, SD: Standard deviation, 95%CI: 95% confidence interval, WOSI: Western Ontario Shoulder Instability Index, MCID: Minimal clinically important difference

## Discussion

The aim of this retrospective study was to compare the clinical and functional outcome of Bankart repair using all-suture anchors with metal anchors with a minimum two-year follow-up. We found no difference in clinical and functional outcomes between the groups. Re-dislocation rates were similar, although the rates were too small for comparison.

All-suture anchors, introduced in 2010^21^, are among the latest group of anchors to be used clinically, while metal anchors were the first to be used for arthroscopic Bankart repair^4,14^. There are certain disadvantages of metal anchors which led to the evolution of different anchor materials over a period of time, yet they still remain popular and are less expensive compared to other anchor devices^5,15,19^.

All-suture anchors have the advantage of smaller diameter of anchors, which allows anchors to be placed as close as 2mm to each other, besides the advantage of less glenoid bone removal^8^. Also, this allows the surgeon to place more than three anchors if he feels the need for the same, for more secure fixation, although there is a risk of glenoid rim fracture in such cases^22^. An average of three all-suture anchors were used per case in our study. The small size of the anchor also makes them useful in revision cases. In addition, the curved drill sleeve allows easier and lower placement of anchor on the antero-inferior glenoid rim^21^. These anchors do not interfere with post-operative MRI, if required, unlike metal anchors.

Different mechanical studies on all-suture anchors, have reported comparable results to conventional anchors (biocomposite, bioabsorbable and PEEK anchor)^7,9,23^. Only a few clinical studies have reported results of all-suture anchor in arthroscopic Bankart repair. Willemot *et al* in a study on 20 patients involving patients requiring Bankart repair, SLAP repair or both with a minimum 1 year follow-up reported satisfactory clinical results using all-suture anchors^6^. Gul *et al* in their study on 62 patients (including 25 patients with concomitant SLAP) with a minimum follow-up of 24 months reported 91.9% good to excellent results and a re-dislocation rate of 8.1 % using double-loaded all-suture anchor in cases of anterior shoulder instability^24^. Lee *et al* in a comparative study of the all-suture anchor with biodegradable suture anchor in a group of 67 patients reported comparative clinical outcomes and post-operative stability between the groups with a minimum two-year follow-up. They used both 1.3 and 1.8mm all-suture anchors in their study^10^.

In our study, we used only a 1.4mm all-suture anchor and found significant improvement in post-operative ROWE and WOSI scores with re-dislocation in 2 out of 41 patient (4.8%). This was in concurrence with previously reported results. We included cases of arthroscopic Bankart repair only and excluded cases requiring concomitant SLAP, posterior labral repair or remplissage.

Concerns have been raised about post-operative tunnel enlargement or large cyst formation in cases of the all-suture anchors but clinical implications of the same as far as instability repair is concerned are not yet known^6,10,21,22,25^. The deployment of the all-suture anchor is dependent on the adequate pulling of threads for proper bunching of the anchor. Dwyer *et al*^7^ in their study on the comparison of maximum load to failure and tensile displacement concluded that a pre-tensioning of the all-suture anchor with force equivalent of 60 N eliminated the difference in load to failure which was there between the handset and pre-tensioned all-suture anchor and the screw in anchor. Therefore, proper pre-tensioning of the all-suture anchor as per manufacturer guidelines is necessary to prevent anchor failure.

Metal anchors though the oldest anchor type, have slowly been replaced by bioabsorbable, biocomposite, PEEK and recently by the all-suture anchor, though all have their unique set of associated complications. Still, metal anchors have retained their usage due to their cost-effectiveness.

Metal anchors have been compared with other anchors previously. Tan *et al*^14^ compared the results of arthroscopic Bankart repair using metal and bioabsorbable anchor on 124 patients with a mean follow-up of 2.6 years and reported comparable results. Milano *et al*^18^ in a similar study on 70 patients with a minimum follow-up of 2 years reported comparable clinical results and a recurrence rate of 3% in the metal group and 6% in the bioabsorbable anchor group. Kocaoglu *et al*^17^ in another study on the comparison of the knotted metal anchor with knotless PEEK anchor on 38 patients with a mean follow-up of 40 months reported comparable clinical outcomes and a similar recurrence rate of 5.6%.

Nagakawa *et al* in a study on anterior glenoid rim fractures after arthroscopic Bankart repair in 129 cases each of metal anchor and all-suture anchor, reported a recurrence rate of 14.8% in the metal group and 17.8% in the all-suture anchor group^22^.

In the present study, we used only 2.8mm titanium metal anchors (knotted) and found significant improvement in post-operative Rowe and WOSI scores at a minimum follow-up of 2 years, with re-dislocation in 3 out of 47 patients (6.3%). This is comparable to previously published reports^19^.

Further in our study, there was no significant difference in the clinical and functional outcome measures as well as recurrence rate between the all-suture anchor and metal anchor groups. Independently both the anchors showed significant improvement in clinical and functional outcomes from pre-operative levels.

Uluyardımcı *et al* in a recent study on 67 patients reported similar outcomes in the mid-term follow-up for all-suture and metal suture anchors for arthroscopic Bankart repair^26^.

To our knowledge, this is the largest cohort study on the comparison of clinical and functional outcomes of 1.4mm all-suture anchors with any other anchor with a minimum 2-year follow-up for arthroscopic Bankart repair.

There are certain limitations of this study. This is a retrospective study. The cases were done by different surgeons. There is a difference in the duration of follow-up of the two anchor types. It was due to the fact that previously metal and other suture anchors were in use. All suture anchors were last to be introduced, it took some time to gain acceptance among surgeons and its number gradually increased over a period of time. We have used only one type of all-suture anchor and different all-suture anchors may behave differently. While this uniformity improves internal validity and is a strength of the present study, the results may not be similarly applicable to other all-suture anchors. Barber *et al*^27^ and Ruder *et al*^28^ reported different biomechanical properties of different all-suture anchors. Another limitation is that we don’t perform MRI or computed tomography of post-operative asymptomatic patients as a routine, therefore we cannot comment on labral healing, bone reaction or any anchor hole dilatation or cyst formation. A prospective study with a bigger sample size and longer follow-up, comparing different types of all-suture anchors and their comparison with different types of anchors may be beneficial in determining the clinical and functional efficacy of all-suture anchors and defining its place in the line-up of different anchors available for arthroscopic Bankart repair.

## Conclusion

In conclusion, all-suture anchor showed comparable clinical and functional results as the metal anchor for arthroscopic Bankart repair at two-year follow-up.
